# The acetabular roof reinforcement plate for the treatment of displaced acetabular fractures in the elderly: results in 59 patients

**DOI:** 10.1007/s00402-021-03829-9

**Published:** 2021-04-11

**Authors:** Dietmar Krappinger, Herbert Resch, Richard A. Lindtner, Johannes Becker, Marian Mitterer, Thomas Freude

**Affiliations:** 1 Department of Trauma Surgery, Medical University of Innsbruck, Innsbruck, Austria; 2 Department of Orthopaedics and Traumatology, Paracelsus Medical University Salzburg, Muellner Hauptstrasse 48, 5020 Salzburg, Austria; 3 Department of Trauma Surgery, BG Unfallklinik Murnau, Murnau, Germany

**Keywords:** Acetabular fracture, Osteoporosis, Elderly, Full weight bearing, Reinforcement ring, Antiprotrusion cage, Total hip arthroplasty

## Abstract

**Introduction:**

Open reduction and internal fixation is considered the gold standard of treatment for displaced acetabular fractures in younger patients. For elderly patients with osteoporotic bone quality, however, primary total hip arthroplasty (THA) with the advantage of immediate postoperative mobilization might be an option. The purpose of this study was to evaluate the clinical and radiological outcomes of surgical treatment of displaced osteoporotic acetabular fractures using the acetabular roof reinforcement plate (ARRP) combined with THA.

**Materials and methods:**

Between 2009 and 2019, 84 patients were operated using the ARRP combined with THA. Inclusion criteria were displaced osteoporotic fractures of the acetabulum with or without previous hemi- or total hip arthroplasty, age above 65 years, and pre-injury ability to walk at least with use of a walking frame. Of the 84 patients, 59 could be followed up after 6 months clinically and radiographically. Forty-nine (83%) were primary fractures and 10 (17%) periprosthetic acetabular fractures.

**Results:**

The mean age was 80.5 years (range 65–98 years). The average time from injury to surgery was 8.5 days (range 1–28). Mean time of surgery was 167 min (range 100–303 min). Immediate postoperative full weight bearing (FWB) was allowed for 51 patients (86%). At the 6-month follow-up, all 59 patients except one showed bony healing and incorporation of the ARRP. One case developed a non-union of the anterior column. No disruption, breakage or loosening of the ARRP was seen. Additional CT scans performed in 18 patients confirmed bony healing. Twenty-six patients (44%) had regained their pre-injury level of mobility. Complications requiring revision surgery occurred in 8 patients. Five of them were suffering from a prosthetic head dislocation, one from infection, one from hematoma and one from a heterotopic ossification.

**Conclusions:**

The ARRP has proven to provide sufficient primary stability to allow for immediate FWB in most cases and represents a valuable option for the surgical management of displaced acetabular fractures in this challenging patient group.

## Introduction

Open reduction and internal fixation (ORIF) is considered the gold standard of treatment for displaced acetabular fractures. The prerequisites for a favorable outcome after ORIF, however, are anatomical reduction and maintenance of reduction until healing. Over the last years the number of patients with osteoporotic acetabular fractures has increased with fractures commonly caused by low energy trauma from a ground standing position [1, 2]. While the typical fracture pattern in younger patients involves the posterior column and posterior wall, the typical fracture pattern in older patients involves the anterior column and the quadrilateral plate (QLP) with concomitant medial dislocation of the femoral head [1, 3, 4]. Fractures in this age group are frequently complex and comminuted with superomedial dome impaction and femoral head lesions [5, 6]. Whereas outcomes after ORIF usually are satisfying in younger patients, outcomes with ORIF alone have been mixed in elderly patients [7, 8]. Subcortical impaction makes anatomic reduction difficult to achieve with the risk of postoperative arthritis and the necessity for secondary total hip arthroplasty (THA) [6, 9–13]. As elderly patients often suffer from several comorbidities and a limited physiological tolerance, a long surgical procedure and a subsequent limited mobility due to restricted weight bearing represent considerable health risks. Due to the special features of osteoporotic acetabular fractures, the desire for primary implantation of a THA has increasingly arisen in recent years [9, 11, 13–15]. The question of stable anchorage of the implant in the fractured acetabulum was in the focus of interest. Authors recommended a cabling reinforcement technique [16], an antiprotrusion cage with additional plating [9, 11, 17, 18] or a two-incision approach technique [13].

The acetabular roof reinforcement plate (ARRP) presented in this study was designed with the intention to achieve a stable fixation that allows full weight bearing (FWB) immediately after surgery without any additional fixation technique. The stability is achieved by an angular stable anchoring technique in the intact iliac bone. The goal of this study is to present the clinical and radiological results of a series of 59 patients with displaced osteoporotic acetabular fractures who were treated with the ARRP and hip arthroplasty.

## Materials and methods

This retrospective study was approved by the local ethics committee and no concerns were raised regarding the use of the ARRP. From 2009 to 2019, eighty-four patients with displaced fractures of the acetabulum were treated with the ARRP as an antiprotrusion cage [19, 20]. Eighty-three were acute fractures and one a non-union after open reduction and internal fixation (ORIF). All 84 patients except 3 were treated in two level-one trauma centers. Inclusion criteria for the insertion of this implant were a displaced acetabular fracture with or without a previous hemi- or total hip arthroplasty, age above 65 years, osteoporotic fracture as identified by a low-energy trauma such as a ground-level fall, significant marginal impaction and pre-injury ability to walk at least with use of a walking frame.

Of the 84 patients, 11 had died due to cardiac failure within the first 6 months after surgery. Another 13 patients were seen only at the 3 months but not at the 6-month follow-up visit and one patient was excluded due to failed former osteosynthesis, leaving 59 patients for full clinical and radiographical examination after 6 months (Table 1). Thirty-four were males and 25 females. The mechanism of injury included a simple ground level fall in 51, a level fall (tractor, ladder) in 3, a ski accident in 3 and a bicycle accident in 2 patients.

**Table 1:** Overview of the 59 patients with displaced acetabular fractures treated with the ARRP and THA

Patient	Age	Sex	Injury mechanism	Type	Classification	Days to surgery	Surgical time (min)	Hb preop	Hb postop	Revisions
1	88	F	Fall	Periprosth	Transv	8	140	11.7	9.6	
2	88	M	Fall	Primary	Ant col	14	115	14.8	12.6	Dislocation
3	84	M	Level fall	Primary	ACPHT	13	145	12.4	10.6	
4	86	M	Fall	Primary	Transv	13	100	11.0	10.9	
5	76	F	Fall	Periprosth	Transv	7	149	11.8	8.5	
6	80	F	Fall	Primary	ACPHT	3	230	11.0	10.8	
7	92	F	Fall	Primary	ACPHT	2	144	12.4	9.7	
8	80	F	Fall	Primary	Both col	6	141	12.6	11.2	
9	73	M	Ski	Primary	Both col	4	230	9.9	8.6	
10	80	M	Fall	Primary	ACPHT	5	127	10.4	11.0	Dislocation
11	83	F	Fall	Primary	Both col	7	260	11.1	9.3	Hematoma
12	87	F	Fall	Primary	ACPHT	3	163	11.3	10.1	
13	81	M	Fall	Primary	ACPHT	9	149	11.8	9.6	
14	79	M	Level fall	Primary	ACPHT	5	158	12.4	9.5	
15	82	F	Fall	Primary	Ant col	16	139	12.5	9.5	
16	79	F	Fall	Primary	ACPHT	11	194	12.3	9.9	
17	71	F	Fall	Primary	ACPHT	12	213	11.0	11.4	
18	94	F	Fall	Primary	Both col	4	242	12.1	11.4	Dislocation
19	70	M	Fall	Primary	ACPHT	3	165	11.4	8.1	Dislocation
20	65	F	Fall	Primary	Transv	7	192	12.5	11.5	
21	85	F	Fall	Primary	ACPHT	7	190	12.1	10.1	
22	76	M	Ski	Primary	Both col	5	223	11.6	9.7	
23	70	M	Ski	Primary	Both col	1	303	12.1	9.2	
24	88	M	Fall	Primary	ACPHT	2	140	10.4	10.0	
25	88	M	Fall	Primary	ACPHT	6	130	11.3	10.1	
26	76	M	Bicycle	Primary	Both col	5	210	11.2	10.9	
27	81	M	Fall	Primary	ACPHT	3	247	11.0	9.5	
28	65	F	Fall	Periprosth	Transv	26	269	10.3	8.5	
29	68	M	Fall	Primary	ACPHT	17	131	10.5	8.2	
30	81	F	Fall	Primary	ACPHT	7	179	12.2	11.4	
31	85	M	Fall	Primary	ACPHT	8	147	11.8	8.3	
32	84	F	Fall	Periprosth	Transv	9	107	10.7	9.4	
33	95	M	Fall	Primary	ACPHT	3	116	13.9	9.9	
34	87	M	Fall	Primary	ACPHT	4	129	10.5	8.6	
35	69	F	Fall	Primary	T-type	2	175	10.2	8.0	
36	74	M	Fall	Periprosth	T-type	8	148	14.2	10.8	
37	71	M	Level fall	Primary	ACPHT	5	130	13.5	8.8	
38	76	M	Fall	Primary	Both col	4	112	9.8	6.8	HO
39	69	M	Bicycle	Primary	Both col	10	123	10.6	8.4	
40	67	M	Fall	Primary	T-Type	16	189	10,2	9.2	
41	83	M	Fall	Primary	Transv	23	166	10.3	9.6	
42	83	F	Fall	Primary	Ant col	28	235	10.4	9.1	
43	88	F	Fall	Primary	Transv	6	120	11.0	9.2	
44	79	M	Fall	Primary	ACPHT	14	160	11.6	10.3	
45	73	M	Fall	Periprosth	ACPHT	10	105	10.7	9.5	
46	73	M	Fall	Primary	Transv	3	202	12.9	10.9	
47	84	F	Fall	Primary	Both col	18	162	11.8	8.9	
48	86	F	Fall	Primary	ACPHT	6	142	10.0	8.8	
49	86	M	Fall	Periposth	Transv	4	173	12.6	9.8	
50	98	M	Fall	Primary	ACPHT	9	168	10.2	7.1	
51	69	M	Fall	Primary	ACPHT	24	210	10,4	7.8	
52	89	F	Fall	Periprosth	T-type	2	122	12.2	9.2	Dislocation
53	75	F	Fall	Periprosth	Transv	9	174	10.3	7.9	Infection^a^
54	90	M	Fall	Periprosth	Transv	8	142	11.1	8.9	
55	65	F	Fall	Primary	Transv	10	133	11.3	8.0	
56	95	M	Fall	Primary	Both col	4	156	10.2	8.2	
57	84	F	Fall	Primary	post wall	4	168	13.3	9.5	
58	91	M	Fall	Primary	Transv	9	153	11.2	8.4	
59	83	M	Fall	Primary	Transv	8	178	11.3	7.4	

*Hb* hemoglobin level, *HO* heterotopic ossification

^a^Although suffering a complication requiring revision surgery, this patient was unable to undergo revision surgery due to her poor general health status

The fractures were classified according to Letournel and Judet [21]. Twenty-five were anterior column posterior hemitransverse fractures (ACPHT), 15 were transverse fractures, 10 both-column fractures, 4 T-shaped fractures, 3 fractures of the anterior column + QLP, 1 posterior wall fracture and 1 was an anterior column fracture.

Forty-nine patients presented with a displaced primary fracture and 10 with a displaced periprosthetic acetabular fracture after a previous hemiarthroplasty (4 patients) or total hip arthroplasty (6 patients).Of the 10 patients with periprosthetic acetabular fractures, 6 had a transverse fracture, 2 a T-type fracture, 1 an ACPHT with disruption of the QLP and 1 a fracture of the QLP without fracture of the columns.

### The ARRP

**Fig. 1:**
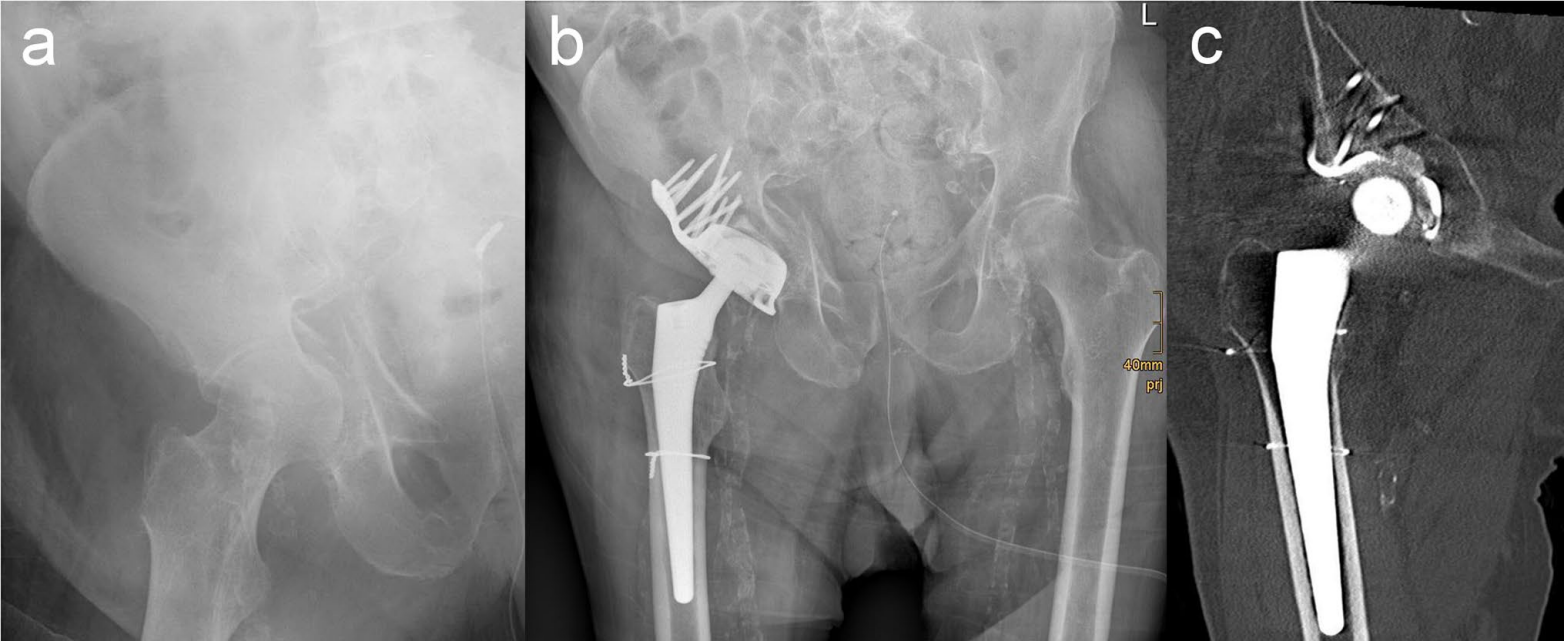
**a** X-ray of a displaced ACPHT fracture. **b** Ap view of the hip joint with ARRP and THA 6 months after surgery. **c** CT imaging confirmed bony healing and osseous incorporation of the ARRP. Uneventful healing of the iatrogenic femoral fracture

For the first 29 patients, the custom-built Acetabulum Roof-Reinforcement Plate (ARRP) 3.5 (DePuy Synthes, Bettlach, Switzerland) was used. For the second series the same implant was used but now certified with a CE mark at least in the last years (41medical AG, Bettlach, Switzerland). The implant, which is an antiprotrusion cage, has an outer diameter of 50 mm and an inner diameter of 48 mm. It is designed for cemented cups with a diameter of 44–48 mm. On the top side, the ring of the implant is extended by a fin which holds 8 angular stable 3.5 mm screws. The design of the holes for the angular stable screws is such that the screws are aiming in divergent directions to increase primary stability. The implant comes in one size with different versions of the fin for the right and left hip. Based on anatomical studies the fin is shaped to fit the acetabular roof and the anterior and middle part of iliac bone. The ring itself holds another seven 3.5 mm holes but experience has shown that only the upper 2 or 4 were important as these screws go into the acetabular roof providing additional stability. Screws placed in the fractured anterior or posterior columns were without any benefit for stability and have been left (Fig. 1b).

### Surgical technique

The surgical technique has already been published [19, 20], and therefore, only a brief description is given here. Under general anesthesia the patient is placed in supine position. Classic lateral [22] or anterolateral approaches for hip replacement are used. In our hands the anterolateral approach (Watson-Jones) approach is preferred as it gives very good access to the acetabular roof and the adjacent iliac bone. After opening the capsule by T-shaped incision the femoral neck is osteotomized. The following steps are described for a primary fracture. The entire capsule is removed in order to provide good exposure of the acetabulum. The cartilage is removed with a spoon and the socket is reamed starting with a 44-mm reamer which goes up step by step to 52 mm. The anterosuperior aspect of the acetabular roof and the adjacent iliac bone is exposed by about 5 cm for positioning the fin. The roof reinforcement plate is introduced without an attempt of prior reduction of the fracture. All holes of the fin and the three upper holes of the ring are used for fixation with angular stable screws to the iliac bone. In most of our cases the anterior and posterior ring holes were not used as fixation of the anterior and/or posterior column seems not to be necessary. In case of an anterior column fracture the fin is placed in the middle of the acetabular roof in order to avoid the fracture line. The femoral head is used for bone grafting on the bottom of the acetabulum to improve bony healing and prevent cement leakage into the pelvis. In case of a periprosthetic acetabular fracture where no femoral head is available, a Prolene Mesh-graft (Ethicon, Johnson & Johnson Medical, Norderstedt, Germany) is fixed with a number of sutures to cover the ring’s inner aperture in order to prevent cement leakage into the pelvis. A cup of 44–48 mm diameter is cemented into the cage. Subsequently the femoral component is implanted.

### Blood transfusion management

According to the individual hospital blood transfusion regime in the two centers the hemoglobin levels were measured pre- and postoperatively. Patients with hemoglobin level less than 10 g/dl and a venous oxygen saturation (ScvO2) below 80% received intraoperative blood transfusion. Patients with hemoglobin levels below 8 g/dl received blood transfusion as well. Same was with symptoms of anemia in the postoperative phase.

All of the patients received Meloxicam© 7.5 mg twice a day or a comparable prophylaxis against heterotopic ossification for 7 days postoperative.

As soon as the patients were able to get up, FWB with the use of a walking frame was started. Only in fractures with involvement of the iliac bone above the acetabular roof partial weight bearing was recommended for the first 3 weeks. In all these suspicious cases a CT scan was performed in order to check the position of the screws.

### Statistical analysis

This study represents a descriptive analysis of our selected patient cohort. Data are presented as means and percentages.

## Results

The average age of all 59 patients was 80.5 years (range 65–98). The average age of the 10 patients with periprosthetic acetabular fractures was 84.6 years (range 73–96). The mean time between accident and surgery was 8.8 days (range 2–28 days). The mean time of surgery was 167.2 min (range 100– 303 min). The average preoperative hemoglobin level was 11.5 g/dl and decreased by a mean of 2.0 g/dl to the average postoperative level of 9.5 g/dl (Table 1).

In 50 patients (85%), early mobilization with FWB was allowed, whereas in 9 patients (15%) only restricted weight bearing was permitted. The reason for restricted weight bearing was an additional femur fracture in one and an undisplaced acetabulum fracture of the other side in another patient. Of the remaining 7 (12%) patients, 6 had a true both column fracture, and one a very high transverse fracture above the acetabulum roof with destruction of the roof and the adjacent iliac bone. In all these cases a postoperative CT scan was performed confirming that only a few screws had found good purchase in stable iliac bone, which was the reason for allowing only partial weight bearing (PWB) for the first 3 weeks.

Of the 59 patients, 26 (44.1%) had regained their pre-injury level of mobility. Fifteen regained full mobility without walking aids, 38 independent mobility with walking aids, and 6 did not reach independent mobility (Table 2).

**Table 2:** Postoperative mobilization and level of mobility of the 60 patients

Patient	Postoperative mobilization	Walking aid before fracture	Walking aid at 6-month follow-up	Pre-injury level of mobility regained?
1	FWB	Yes	Not mobile	No
2	FWB	No	Yes	No
3	FWB	No	No	Yes
4	FWB	No	Yes	No
5	FWB	Yes	Yes	Yes
6	FWB	No	Yes	No
7	FWB	Yes	Yes	Yes
8	FWB	No	Yes	Yes
9	FWB	No	No	Yes
10	FWB	Yes	Yes	Yes
11	FWB	No	Yes	No
12	FWB	Yes	Yes	Yes
13	FWB	No	Yes	No
14	FWB	No	Yes	No
15	FWB	No	Yes	No
16	FWB	No	Yes	No
17	FWB	No	Not mobile	No
18	FWB	No	Yes	No
19	FWB	No	Not mobile	No
20	FWB	No	Yes	No
21	PartialWB	No	Yes	No
22	PartialWB	No	Yes	No
23	PartialWB	No	Yes	No
24	FWB	No	No	Yes
25	FWB	No	No	Yes
26	PartialWB	No	Yes	No
27	FWB	No	Yes	No
28	PartialWB	No	No	Yes
29	FWB	No	Not mobile	No
30	FWB	No	Yes	No
31	FWB	Yes	Not mobile	No
32	FWB	No	No	Yes
33	FWB	Yes	Yes	Yes
34	FWB	Yes	Yes	Yes
35	FWB	Yes	Yes	Yes
36	FWB	No	No	Yes
37	FWB	No	Yes	No
38	FWB	No	Yes	No
39	FWB	No	Not mobile	No
40	FWB	No	No	Yes
41	FWB	Yes	Yes	Yes
42	FWB	No	Yes	No
43	FWB	Yes	Yes	Yes
44	FWB	Yes	No	Yes
45	FWB	No	No	Yes
46	FWB	No	Yes	No
47	FWB	No	No	Yes
48	FWB	No	Yes	No
49	FWB	Yes	Yes	Yes
50	FWB	No	No	Yes
51	PartialWB	Yes	Yes	No
52	FWB	No	No	Yes
53	FWB	Yes	No	Yes
54	PartialWB	Yes	Yes	No
55	PartialWB	Yes	Yes	Yes
56	PartialWB	Yes	Yes	No
57	FWB	No	No	Yes
58	FWB	Yes	Yes	No
59	FWB	Yes	Yes	No

*FWB* full weight bearing, *PartialWB* partial weight bearing for the first 3 weeks postoperatively

Radiographically all fractures were healed except one. In this case with a T-type fracture, the anterior column developed a non-union, whereas the posterior column was healed and the ARRP was incorporated. The non-union was confirmed by CT scan and had to be re-operated with plate fixation about 10 months after the index surgery due to pain when walking. There was no disruption, breakage or loosening of the ARRP.

In 18 patients, an additional CT scan was performed between 3 and 6 months after surgery. Fracture healing and ARRP incorporation was confirmed in all of these cases and no loosening signs were found, except the one mentioned above.

In the 10 patients with periprosthetic acetabular fracture, no bone grafting was performed at the time of surgery due to the lack of a femoral head. Despite the lack of bone grafting, perfect bony healing and incorporation of the ARRP was seen in all cases. No loosening signs were detected. Four of the 10 patients regained their previous level of mobility, 5 did not reach the same level but regained independency and one lost independency. In only one patient allograft was used to prevent cement leakage into the pelvis, whereas in 7 a Prolene mesh-graft (Johnson & Johnson) was used to close the inner aperture of the ARRP.

### Complications and revisions

In five patients, recurrent hip dislocation occurred and required revision surgery with changing of the cup. However, in all five cases the ARRP was stable incorporated and could be left in place. Early infection was seen in one patient suffering from diabetes. Due to the poor general condition of the patient no further surgical interventions could be performed. In two cases with periprosthetic acetabular fracture, cement leakage into the pelvis was observed on postoperative X-rays. This was due to the lack of bone graft on the bottom of the ARRP when the cup was cemented. In both cases the cement could be left as there were no clinical consequences. In two patients, periarticular heterotopic ossification was seen on the follow-up X-rays but surgical removal was necessary in only one case. In one case a postoperative hematoma had to be evacuated surgically. In total, eight patients (13,6%) suffered a complication requiring revision surgery.

At the 3-month follow-up, 76 of the 84 patients were examined radiographically. In all 76 patients, the fractures were consolidated and the ARRP was incorporated except for the one mentioned above with the non-union of the anterior column. In another patient the cemented cup has shown loosening signs but not the ARRP. Revision was not performed in this case.

## Discussion

Osteoporotic acetabular fractures are increasingly common due to the growing elderly population [2]. Non-operative treatment is considered the primary choice of treatment for nondisplaced acetabular fractures in older patients [23]. However, fractures in this age group are much more commonly comminuted and complex with marginal impaction, femoral head damage, fracture of the QLP and the presence of a superomedial dome impression (gull sign) [5, 15, 24, 25].

For most acetabular fracture patterns, ORIF is still considered the gold standard of surgical treatment. In order to avoid the rather invasive and time-consuming ilioinguinal approach, minimally invasive techniques were introduced more recently [4, 26–29]. Ruchholtz et al. [29] presented a new two-incision minimally invasive technique (TIMI) with the promising results. However, their case series is difficult to compare with our series as only 14 of the 26 (54%) patients were older than 65 years. Another minimally invasive technique is the so-called Pararectus approach proposed by Keel et al. [28] who also reported with very promising results. In this series, however, the average age was 62 years and only 48% of the patients were older than 60 years. As the average age of our series was 80.5 years comparison is difficult. Another minimal invasive technique using the modified Stoppa approach combined with the first window of the ilioinguinal approach was presented recently together with the introduction of a new plate, the so-called acetabulum wing plate [4]. This custom-made plate is especially designed for the fixation and stabilization of the QLP counteracting the force of the femoral head. Again the results obtained with this technique were promising, but the average age of the published series of 12 patients was 62.5 years and thus results may not be directly comparable to those of our series. Another issue is that postoperative weight bearing restrictions and mobilization protocols often were not further specified in these studies.

In order to obtain good results with open reduction and internal fixation (ORIF) anatomic reduction is essential [30–34]. In older patients, subcortical impaction is very common which makes anatomic reduction difficult to achieve [6, 9–13]. In the literature the rate of secondary THA after ORIF alone reaches from 19 to 70% due to postoperative arthritis (Archdeacon et al. 19% [35], Lont et al. 30% [11], Boelch et al. 45% [14], Kreder et al. 54% [15], and Borg et al. 70% [9]).

A major issue with ORIF alone is the long duration of restricted weight bearing from at least 6–12 weeks [16, 36]. Most elderly patients do not comply with the weight bearing restriction resulting in either secondary displacement or permanent immobilization. Even temporary immobilization results in a decrease of bone metabolism [26] and exacerbation of possible comorbidities. Early FWB should therefore be a key part of the management strategy [9, 11, 13, 14, 37].

The high rate of postoperative arthritis after ORIF with the consequence of conversion to secondary THA made several surgeons to change the strategy away from ORIF alone to combined techniques with primary THA and ORIF [13, 14, 37, 38]. Some were using a reinforcement ring [11, 14, 18, 39, 40], and others preferred a two-incision technique with stabilization of both columns [13]. Studies comparing elderly patients treated with ORIF alone and those treated with a combined procedure have shown better outcome for the primary arthroplasty combined with ORIF group [9, 11, 13, 14].

Increasing evidence supports the practice of using only a single surgical approach, especially for this elderly age group. Several authors, for example, promoted a single posterior approach with bridge plating of the posterior column and implantation of an acute THA [5, 37, 40, 41].

**Fig. 2:**
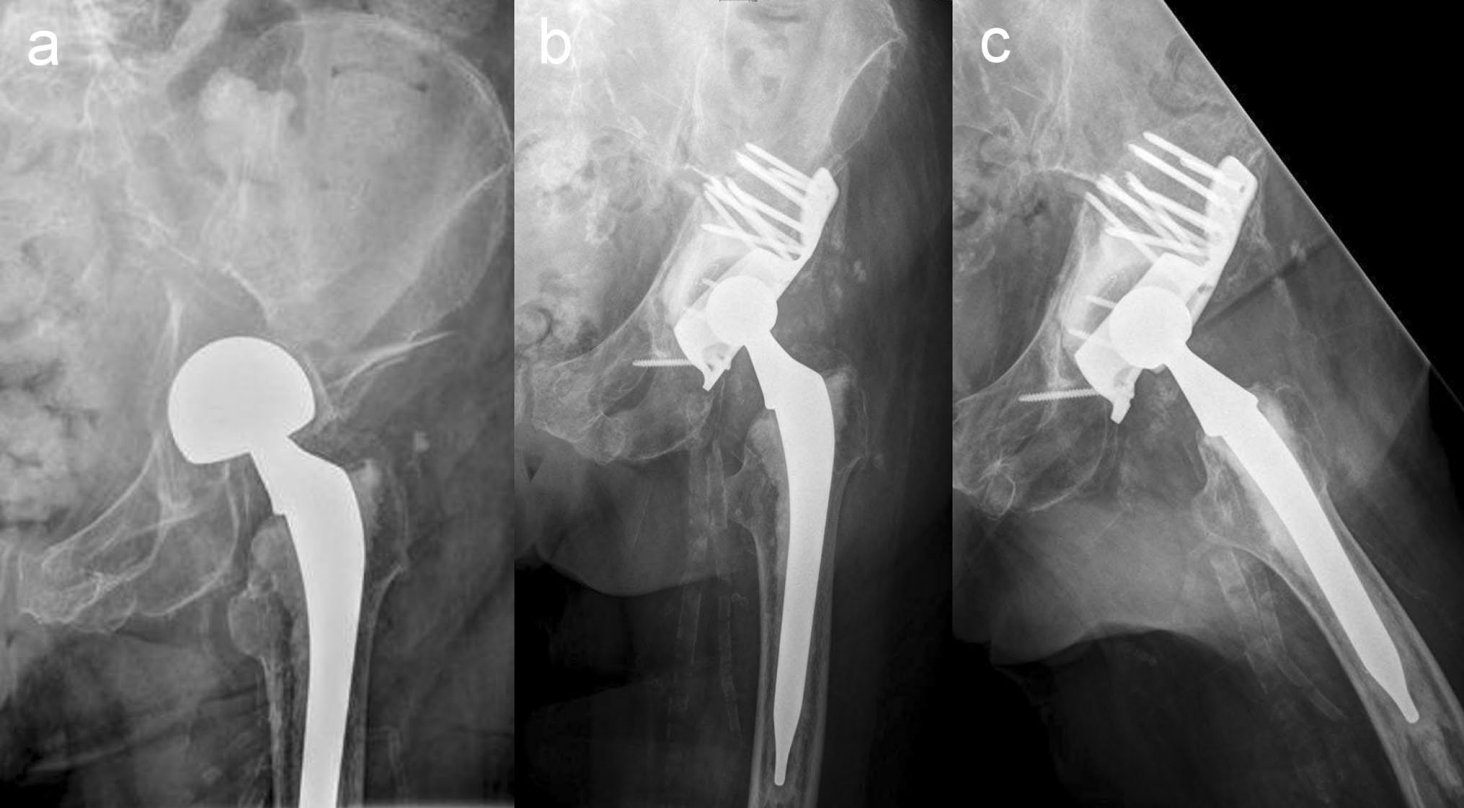
**a** X-ray of a periprosthetic acetabular fracture. **b** Postoperative X-ray of the hip joint with implantation of ARRP and THA. **c** After 6 months fracture healed without bone grafting due to the lack of femoral head

The standard lateral or anterolateral approach to the hip joint can be used for the procedure described in this paper as there is no need for fracture reduction and fixation. The key part of the presented procedure is an antiprotrusion ring called ARRP (41medical, Bettlach Switzerland). The ARRP is characterized by a high primary stability allowing for early FWB in almost all cases [42]. The high primary stability is provided by a high number of 3.5-mm angular stable screws fixed in stable iliac bone (2–4 upper ring screws and 8 screws through the fin). Another reason for the high stability of the ARRP might be caused by the fact that the monoaxial locking screws with different angles of the individual screws with respect to the implant might provide additional purchase in the iliac bone. Despite the fact that almost all patients were allowed for FWB from the first postoperative days, we did not see a single case of ARRP disruption or breakage. A major advantage of this procedure is that no fracture reduction or fixation is necessary. All fractures but one healed and incorporation of the ARRP was observed in all cases (Figs. 1 and 2). In only one patient with a T-type fracture the anterior column developed a non-union, while the posterior column had healed and the ARRP was incorporated. In this case, plating of the anterior column led to bony consolidation. Surprisingly, healing and incorporation of the ARRP also occurred in all periprosthetic acetabular fracture cases in which bone grafting was not possible due to the missing femoral head.

In a recent biomechanical study, the primary stability of ARRP and the Burch Schneider reinforcement ring were compared. According to Culemann et al. [43] we used an anterior column with posterior hemitransverse fracture model. In this study, the high primary stability of the ARRP was demonstrated and was significantly higher than that of the Burch Schneider ring [42].

Fifty (85%) of our 59 patients were allowed immediate FWB, whereas in 9 patients only restricted weight bearing was permitted. In two of these nine patients, the reasons for only toe touch or partial weight bearing were a fracture of the femur shaft which occurred intraoperatively (1 case) and an undisplaced acetabulum fracture of the other side (1 case). Consequently, only seven patients had restricted weight bearing for implant reasons. Six of these seven patients were suffering from a two-column fracture and one from a high transverse fracture with a destroyed acetabulum roof. In all these seven patients, a CT scan confirmed that the screws had not sufficient purchase in stable iliac bone and FWB was thus allowed not before the fourth postoperative week.

Due to the fact that fracture reduction and fixation was not necessary with this technique, the duration of the surgical procedure was only 167 min (range 100–303 min) which is short compared to other studies (Rickman et al. 193 min [13], Boelch et al. 189 min [14], Herscovici et al. 232 min [40]). The average postoperative hemoglobin difference in our case series was 2.0 g/dl, which combined with the average intraoperative transfusion of 1.2 units of blood equals approximately 4.0 g/dl according to Pierson et al. [44] and therefore does not differ from the results for primary THA, which has been reported to be 3.5–4.0 g/dl [45].

Whereas the patients in this series being 80 years old on average, we record 11 deaths within the first 6 months after surgery due to cardiac failure. The youngest of them was 76 years; all others were 85 or older and 5 of them had 90 years or more. Rickman et al. [13] (40) reported a similar mortality rate of 14%, although the average age in their series was 77 years and thus slightly lower.

The overall revision rate within the first 6 months was 12% (7/59 cases) in our series. In addition, one patient suffering from diabetes had developed infection which could not be revised due to the poor general condition of the patient. In five patients, a recurrent dislocation of the prosthetic head occurred with the necessity of reoperation and change of the cup. We believe that poor positioning of the cemented cup inside the ARRP might have caused dislocations. In another patient, a hematoma had to be evacuated surgically. In two patients, periarticular heterotopic ossification was detected on follow-up radiographs but only one of them had to be revised surgically.

Another complication without any clinical consequences was cement leakage into the pelvis in two patients with a periprosthetic acetabular fracture. In these patients, no bone grafting was possible which would have sealed the pathway into the pelvis. Even though this was without any harm to the patients, we started using a Prolene mesh-graft (Ethicon, Johnson & Johnson Medical, Norderstedt, Germany) in periprosthetic fracture cases where no femoral head was available for bone grafting.

Overall, we observed very satisfying results, especially regarding early postoperative mobilization and the pre- to postoperative mobility level. Compared to our study only Rickman et al. [13] presented a higher rate of early FWB which was 100% but with a much smaller number of patients and a slightly lower mean age. They described the postfracture mobility as independent but still mostly requiring walking aids. In our series at the 6-month follow-up, 44% (26/59) regained their pre-injury level of mobility. Fifteen did not need walking aids at all, 38 needed walking aids but were independent mobile and 6 did not reach independent mobility.

A limitation of this study is the retrospective study design, the relatively short follow-up time of 6 months and the loss of 25 patients to follow up. Of these, 11 had died due to preexisting comorbidities and 14 did not come to the 6-month follow-up visit. A further limitation is that not all patients underwent a CT scan 6 months after surgery to analyze fracture healing and to detect potential loosening of the implant. However, all patients underwent at least biplane radiography in addition to the clinical investigation. On the other hand, all patients have been operated with this new implant in trauma centers. In case of any problems with the operated hip, the patients would have been assigned to the department where the operation took place. So far, no complications of this kind have become known and that applies not only to the 59 patients but to all 84 patients.

## Conclusion

The patient group we are dealing with here is very challenging. Specific characteristics such as age, fracture pattern, osteoporosis, and comorbidities require individual decision-making. Based on our results the ARRP designed for the treatment of displaced acetabular fractures with poor bone quality represents an additional option to the spectrum of treatments in this challenging patient group. Owing to the special design, the ARRP provides high primary stability and immediate FWB is possible in almost all cases. The absence of the need for fracture reduction and stabilization shortens operating time and reduces intraoperative blood loss. However, careful patient selection, preoperative planning, and workup are required. The lack of long-term follow-up data is a shortcoming of the AARP, but this data is particularly difficult to obtain in this specific age group.
